# 

**Published:** 1879-07

**Authors:** 


*CONTENTS.*

| | Page | | Page |
|---|---|---|---|
| Theological Theraputics | 1 | Agricultural Review | 23 |
| Early Rising | 3 | Style | 26 |
| Climate for Consumptives | 4 | Music Lessons | 26 |
| Summer Hegira | 5 | The Influence of Temper on Health | 27 |
| Raising Children | 6 | The Loss of Children | 27 |
| Geology and Genesis | 12 | Secret of Eloquence | 28 |
| Hidden Tteasures | 14 | Christianity Aggressive | 28 |
| Checking Perspiration | 17 | Constipation | 29 |
| The Dying | 17 | Diseases of Children | 30 |
| Hygiene | 19 | | |

